# Exosomal CA125 as A Promising Biomarker for Ovarian Cancer Diagnosis

**DOI:** 10.7150/jca.48531

**Published:** 2020-09-14

**Authors:** Zhixiang Chen, Qianxin Liang, Hua Zeng, Qing Zhao, Zhaodi Guo, Rihui Zhong, Manlin Xie, Xiuping Cai, Jing Su, Zhiliang He, Lei Zheng, Kewei Zhao

**Affiliations:** 1 Guangzhou University of Chinese Medicine, Guangdong Guangzhou, 510006, China.; 2 The Third Affiliated Hospital of Guangzhou University of Chinese Medicine, Guangdong Guangzhou, 510378, China.; 3 Department of Clinical Laboratory, Sun Yat-sen Memorial Hospital, Sun Yat-sen University, 510120, China.

**Keywords:** ovarian cancer, exosome, carbohydrate antigen 125, human epididymis protein 4

## Abstract

The main diagnostic indicators of ovarian cancer (OC), including carbohydrate antigen 125 (CA125) and human epididymis protein 4 (HE4), show good sensitivity and poor specificity or vice versa. This study investigated changes in CA125 and HE4 expression and their correlation in serum-derived exosomes of 55 patients with OC (OC group), 33 patients with malignant tumors (non-OC group), and 55 normal controls (NC group). We compared serum and exosomal CA125 and HE4 levels to determine whether their contents in exosomes were elevated. We also compared the diagnostic efficacy of serum HE4, serum CA125, exosomal CA125, and serum HE4+exosomal CA125 in OC using the receiver operating characteristic (ROC) curve. CA125 levels in serum-derived exosomes in all groups significantly increased (*P* < 0.0001) compared with serum CA125 levels. HE4 was undetected in exosomes. The ROC curve showed the following values: serum CA125: 0.9093 (area), 87.27% (sensitivity), and 90.91% (specificity); serum HE4: 0.9302, 83.64%, and 94.55%; exosomal CA125: 0.9755, 94.55%, and 92.73%; and serum HE4+exosomal CA125: 0.9861, 96.36%, and 92.73%. In conclusion, CA125 can be detected at higher levels in exosomes than in serum, significantly improving OC diagnosis sensitivity. The serum HE4+exosomal CA125 combination significantly improves OC diagnostic efficiency.

## Introduction

Ovarian cancer (OC) is one of the deadliest gynecologic malignancies and the fifth-leading cause of cancer deaths worldwide 1. Early OC symptoms are not specific 2. Further, OC shows rapid progress and easy dissemination, so most patients with OC are in the middle or late stage at the time of diagnosis. Therefore, serum tumor markers such as carbohydrate antigen 125 (CA125), human epididymis protein 4 (HE4) and B-ultrasound are commonly used for early screening for OC. However, they are good in terms of sensitivity and poor in terms of specificity, or vice versa 3-5, so it is necessary to identify rapid and effective indicators for early diagnosis of OC.

Exosomes are extracellular vesicles 40-100 nm in diameter. After multivesicular bodies of the endocytic system fuse with the cell membrane, they are released to the outside of the cell as an exocrine secretion 6,7. They occur widely in various body fluids 8 and participate in biological processes such as tumor cell proliferation, metastasis, and drug resistance 9. Exosomes in the serum of patients with tumors contain proteins, messenger RNA (mRNA), microRNA (miRNA), and lipids specific to tumor tissues. The content of some biological components of tumor exosomes increases significantly and has tissue specificity. Therefore, exosomes, as a tumor-specific marker for early OC diagnosis, have good development prospects. In the future, exosomes may provide tumor markers with high specificity and sensitivity for OC diagnosis to reduce the OC mortality rate.

Few studies have reported the correlation between exosomal CA125 and HE4 levels and OC. Therefore, do serum-derived exosomes in OC contain CA125 and HE4 and are their specificity and sensitivity more significant? This study investigated changes in CA125 and HE4 expressions in serum-derived exosomes in patients with OC and their correlation to obtain new observation indexes for the early diagnosis and clinical treatment of OC.

## Materials and Methods

### Sample collection

We collected 143 individuals' serum samples from the Third Affiliated Hospital of Guangzhou University of Chinese Medicine, China, and the Sun Yat-sen Memorial Hospital, China, from May 2019 to January 2020. The inclusion criterion was diagnosis of malignant tumors that met the diagnostic criteria of histopathology. Individuals in the normal control (NC) group had no history of tumor. The exclusion criteria were as follows: (1) complicated with blood system diseases, thrombosis, and hemorrhagic diseases and (2) patients on antibiotics, anticoagulants, hormones, or nitrogen-containing drugs and with massive blood loss before admission that would affect the test results of all indicators. The 143 individuals were categorized into the OC group (*n* = 55; 39 serous OC, 10 ovarian adenocarcinoma, 1 ovarian carcinosarcoma, 1 ovarian clear cell carcinoma, 3 mucinous ovarian carcinoma, and 1 endometrioid ovarian carcinoma), non-OC group (*n* = 33; patients with various malignant tumors including 10 with lung cancer, 6 with gastric carcinoma, 5 with colon cancer, 9 with liver cancer, and 3 with rectal cancer), and NC group (*n* = 55; age-matched individuals with healthy serum). The mean age of the OC group was 55.2 ± 1.6 years; non-OC group, 61.5 ± 2.0 years; and NC group, 53.0 ± 1.3 years. There was no significant between-group difference in age (*P* > 0.05). The study was approved by the ethics committee of our hospital, and all patients provided signed informed consent.

### Exosome extraction

**Figure 1:**
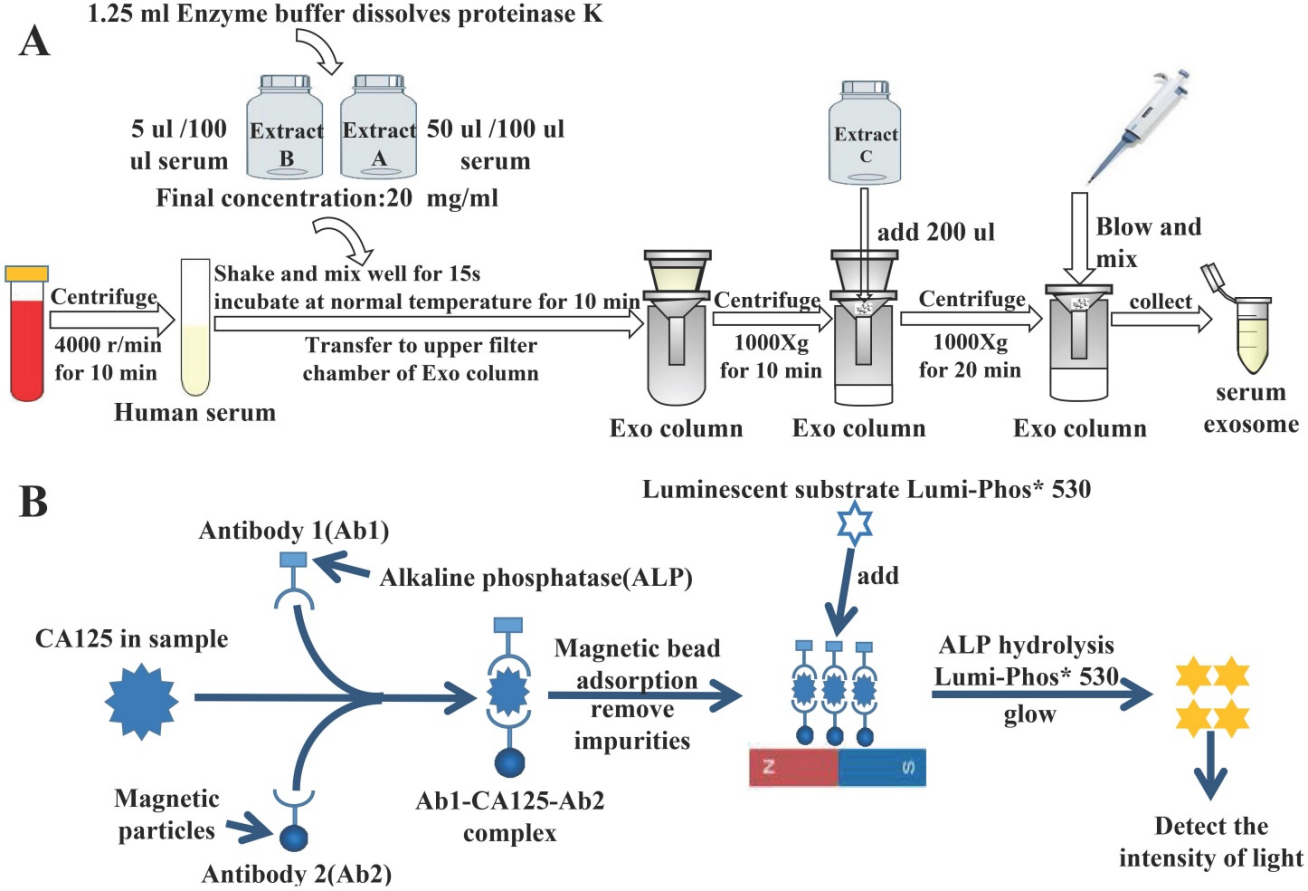
Experimental principle. A) Flowchart of extraction of serum-derived exosomes. B) Specific process of detecting CA125 by chemiluminescence.

Exosomes were extracted from the serum using the extraction procedure specified on the used kit (Guangzhou Supbio Biology Co., Ltd., Guangzhou, China) (Fig. 1A).

### Transmission electron microscopy

We dropped 10 μL of the exosome sample onto a copper mesh and precipitated it for 3 min. The filter paper sucked the floating liquid from the edge. The sample was rinsed with phosphate-buffered saline (PBS), and then stained with phosphotungstic acid. The sample was dried at room temperature for 5 min and observed under a JEM-1200EX transmission electron microscope (Japan Electronics Co., Ltd., Tokyo, Japan), and images were obtained for preservation.

### Nanoparticle tracking analysis

First, the 10 μL of the exosome sample was diluted. Then, internal components and test channels of the Malvern NanoSight NS300 instrument (Malvern Panalytical, Malvern, UK) were cleaned. The instrument absorbed the diluted exosome samples. After injecting, data and images were saved and output.

### Flow cytometry

First, 500 μL of the exosomal sample was taken, 1 mL of PBS was added, and then 20 μL of CD9 and CD63 antibodies were added. After incubation at 37°C for 30 min, the processed sample was ultracentrifuged at 54,000 rpm at 4°C for 24 min. Ultracentrifugation was repeated, the supernatant was removed, 40 μL of PBS was added for resuspension in dark conditions, and the suspension was detected using the Flow NanoAnalyzer (NanoFCM N30, Guangzhou, China).

### Tumor marker detection

First, we collected 3 mL of venous fasting blood from all participants in serum separation tubes and centrifuged it immediately at 3000 rpm for 5 min. The supernatant was collected to measure serum tumor markers. Serum alpha-fetoprotein (AFP), CA125, CA153, CA199, and carcinoembryonic antigen (CEA) levels and exosomal AFP, CA125, CA153, CA199, and CEA levels of the non-OC group were detected using the Beckman DXl800 chemiluminescence immunoassay device (Beckman Coulter Inc., Brea, CA, USA). Serum HE4 levels of the non-OC group were detected using the Roche Cobas 602 analyzer (Roche Diagnostics, Basel, Switzerland). Serum CA125 and HE4 and exosomal CA125 and HE4 levels of the OC and NC groups were also detected using these methods. Fig. 1B shows the specific process of chemiluminescence detection of CA125.

### Statistical analysis

Experimental results were analyzed using the GraphPad Prism 8.0 statistical software. Differences between serum and exosome among the groups (OC, NC, and non-OC) were assessed using Student's *t*-test, and multiple comparisons of all three groups were performed using one-way analysis of variance. A receiver operating characteristic (ROC) curve was constructed to evaluate the diagnostic value of serum CA125, serum HE4, exosomal CA125, exosomal CA125/serum CA125, serum HE4+serum CA125, and serum HE4+exosomal CA125 in OC. A *P*-value of <0.05 was considered statistically significant.

## Results

### Identification of serum-derived exosomes

**Figure 2:**
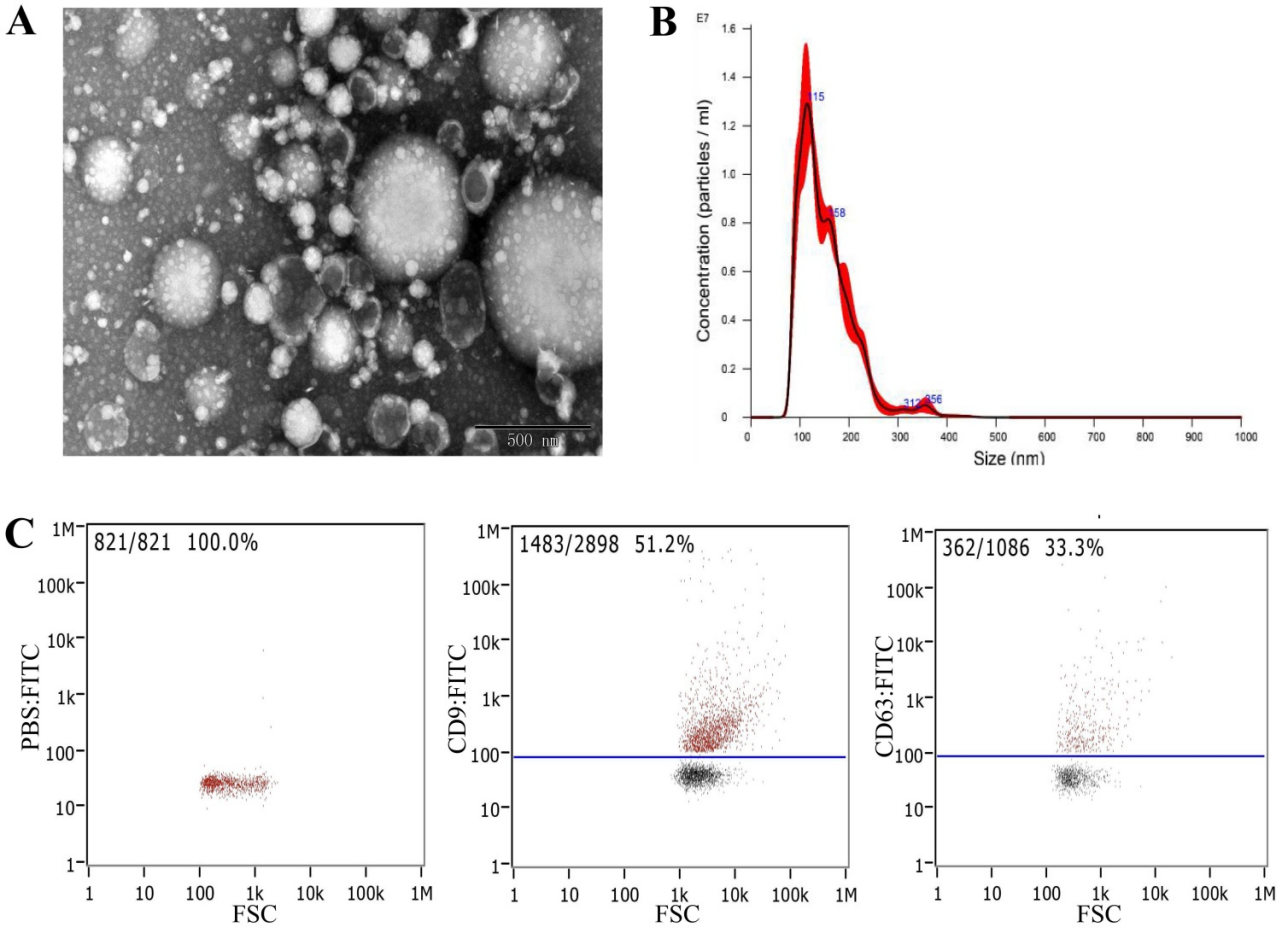
Identification and extraction of serum-derived exosomes. A) Morphology of serum-derived exosomes identified by TEM. B) NTA revealed the size distribution of serum-derived exosomes. C) Expression of the characteristic proteins CD9 and CD63 of exosomes identified by flow cytometry.

The morphology of serum-derived exosomes was observed using transmission electron microscopy (TEM). The results showed that exosomes were round or oval cuplike vesicles with obvious exosomal shape, but the samples contained some impurities (Fig. 2A). Nanoparticle tracking analysis (NTA) was performed to detect the particle size distribution and concentration of the exosomes. The results showed that the concentration was 6.20 × 10^11^ particles/mL, and the particle size distribution was between 0 and 300 nm (Fig. 2B). Flow cytometry showed that the exosomes expressed exosomal surface markers CD9 and CD63 (Fig. 2C). These findings indicated that we had successfully extracted exosomes from serum.

### Detection of tumor markers in three groups of serum and serum-derived exosomes

**Figure 3:**
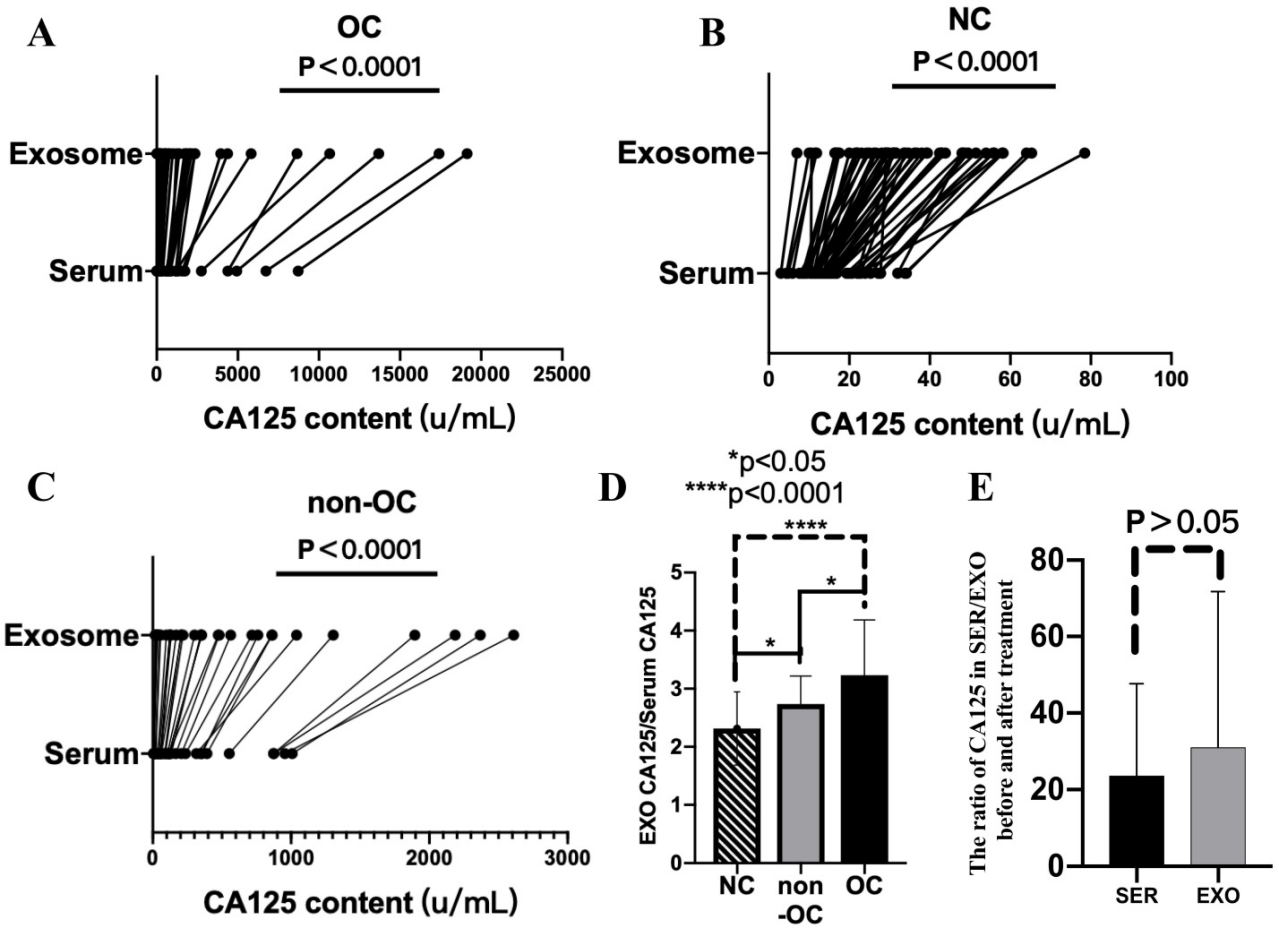
Serum and exosomal CA125 levels of the ovarian cancer (OC), normal control (NC), and non-OC groups. A-C) Difference in CA125 expression between serum and exosomes in the OC (*n* = 55), NC (*n* = 55), and non-OC (*n* = 33) groups. D) Calculation of the exosomal/serum CA125 ratio in the three groups (*n* = 33) to analyze the degree of increase in CA125 level in exosomes. E) Difference in pretreatment/post-treatment serum CA125 levels and pretreatment/post-treatment exosomal CA125 levels in the OC group (*n* = 6).

Next, serum and exosomal CA125 and HE4 levels of the OC, NC, and non-OC groups were detected. Compared with serum CA125 levels, exosomal CA125 levels all significantly increased, and the difference was significant (*P* < 0.0001) (Fig. 3A-C). However, exosomal HE4 levels of the OC, NC, and non-OC groups could not be detected.

The degree of increase in CA125 level in the exosomes was analyzed by calculating the exosomal/serum CA125 ratio in 33 individuals who were randomly selected from each group. The exosomal/serum CA125 ratio was the highest in the OC group, followed by the non-OC group and then the NC group. There were significant differences (*P* < 0.05) among all groups (i.e., OC vs. NC, *P* < 0.0001; OC vs. non-OC, *P* < 0.05; and NC vs. non-OC, *P* < 0.05) (Fig. 3D).

We also calculated the results of pretreatment/post-treatment serum CA125 levels and pretreatment/post-treatment exosomal CA125 levels. Comparison of the calculated results of these two groups revealed that the rate of decrease in CA125 level in serum-derived exosomes was not different from that in serum (*P* > 0.05) (Fig. 3E).

**Figure 4:**
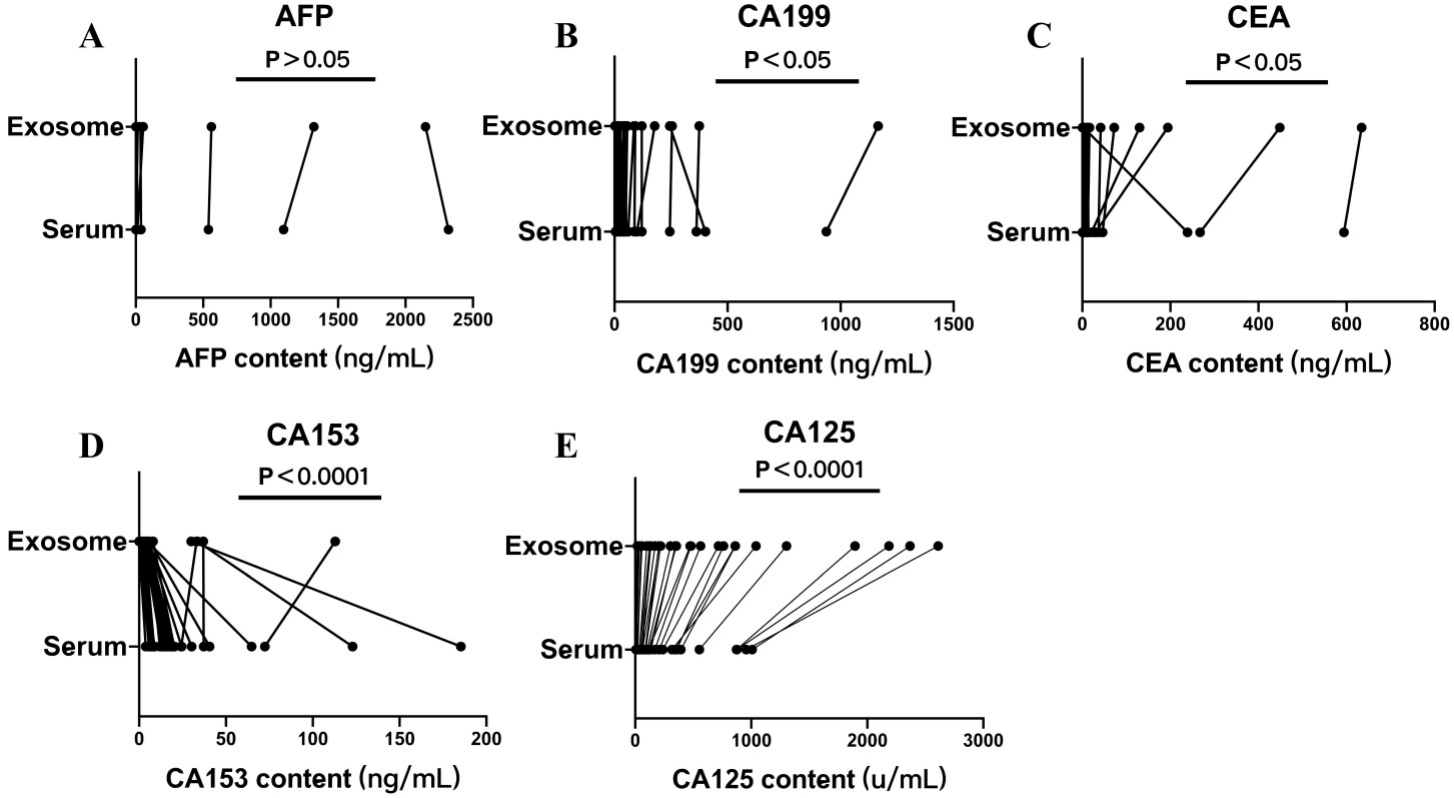
Serum and exosomal AFP, CA199, CEA, CA125, and CA153 levels of the non-ovarian cancer (OC) group. Differences in A) AFP, B) CA199, C) CEA, D) CA153, and E) CA125 levels between serum and exosomes of the non-OC group.

Serum and exosomal AFP, CA125, CA153, CA199, CEA, and HE4 levels of the non-OC group were measured. There was no significant difference between AFP levels in serum and exosomes (*P* < 0.05) (Fig. 4A). The differences between serum and exosomal CA199 and CEA levels were significant (*P* < 0.05) (Fig. 4B, C). The differences between serum and exosomal CA153 and CA125 levels were significant (*P* < 0.0001) (Fig. 4D, E). Compared with serum levels, exosomal CA153 levels showed an overall decreasing trend, whereas exosomal CA199, CEA, and CA125 levels showed a significantly increasing trend.

### Comparison of the diagnostic efficacy of serum HE4, serum CA125, exosomal CA125, and their combinations in OC

**Figure 5:**
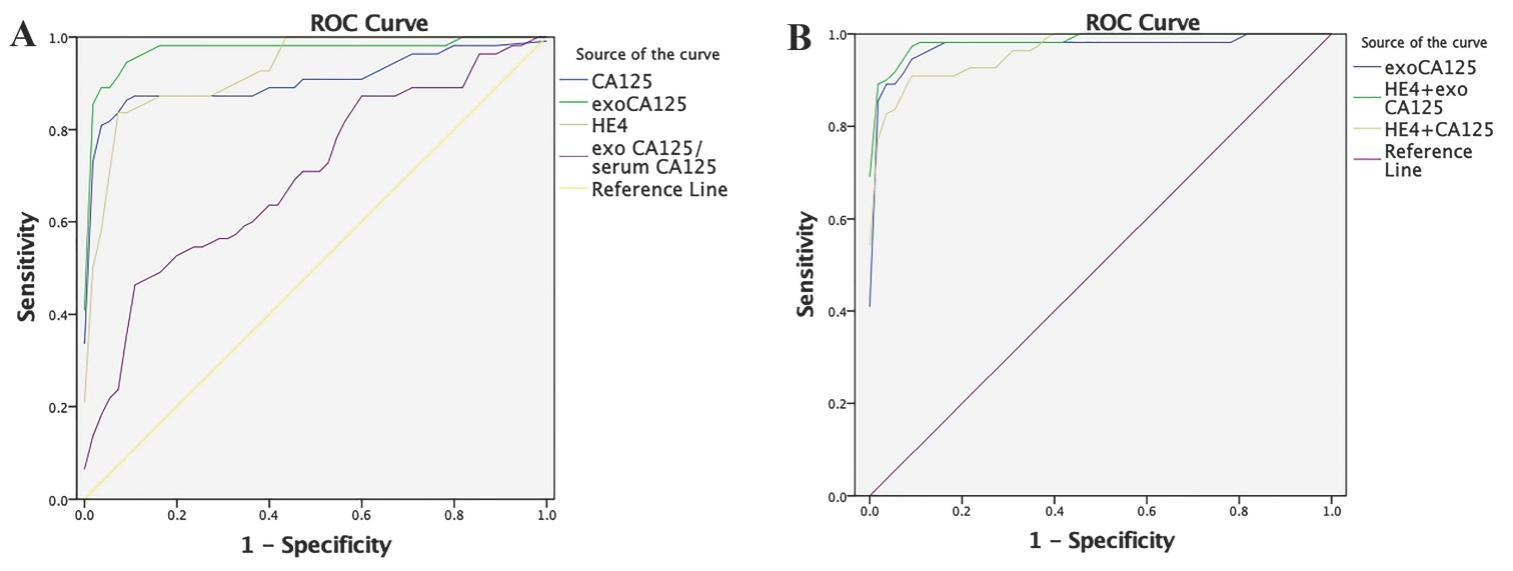
Diagnostic efficacy of OC-related indicators in ovarian cancer (OC) evaluated by the ROC curve. A) Diagnostic efficacy of serum CA125, exosomal CA125, serum HE4, and exosomal/serum CA125 ratio in OC was evaluated by the ROC curve. B) Diagnostic efficacy of exosomal CA125, serum HE4+exosomal CA125, and serum HE4+serum CA125 in OC was evaluated by the ROC curve.

In the OC group, the area under the ROC curve (AUC) of serum CA125 was 0.9093, sensitivity was 87.27%, and specificity was 90.91%, and the AUC of serum HE4 was 0.9302, sensitivity was 83.64%, and specificity was 94.55%. In contrast, the AUC of exosomal CA125 was 0.9755, sensitivity was 94.55%, and specificity was 92.73%. The AUC of the exosomal/serum CA125 ratio was 0.6997, sensitivity was 47.27%, and specificity was 89.09%. Finally, the AUC of serum HE4+serum CA125 was 0.9646, sensitivity was 90.91%, and specificity was 92.73%. Serum HE4+exosomal CA125 had the best effect: the AUC was 0.9861, sensitivity was 96.36%, and specificity was 92.73% (Fig. 5). Table 1 lists these results.

## Discussion

OC is one of the three common malignant tumors of the female reproductive system that poses a serious threat to women's health. Because of the complex endocrine function of the ovary, the early OC symptoms are unspecific, so patients usually miss the optimum treatment window by the time they are diagnosed 10. Recently, the OC incidence rate has increased significantly and patients tend to be younger at diagnosis. Therefore, finding early diagnostic indicators with high specificity and sensitivity can substantially improve the survival rate and prognoses of patients with OC.

Tumor markers, B-ultrasound, and magnetic resonance imaging (MRI) are commonly used for early OC diagnosis in clinical settings. OC diagnosis by B-ultrasound mainly depends on the experience and subjective judgment of the examiner 11,12, who might miss an early diagnosis. Abnormal tumor marker levels are often detectable before changes on MRI, which is more conducive to early OC diagnosis. Different tumor markers have different disadvantages. For example, CA125, a common tumor marker in clinical settings, has high sensitivity in OC. However, it might be elevated in women with highly prevalent benign diseases 13. HE4 is a new serum marker for OC diagnosis 14; it is highly expressed in OC but it has low sensitivity 15. Therefore, the tumor marker level cannot serve as the basis for definite OC diagnosis. However, it plays an important role in early OC diagnosis and is relatively simple, non-invasive, and effective for early screening and diagnosis of OC.

The risk of ovarian malignancy algorithm (ROMA) is calculated by combining CA125 and HE4 levels and dividing it into premenopausal predicted risk probability (PPI) and postmenopausal predicted risk probability (PPII). The specific calculation formulas are as follows:

Premenopausal index (P1) = -12.0 + 2.38 × LN (HE4) + 0.0626 × LN (CA125)

Postmenopausal index (P2) = -8.09 + 1.04 × LN (HE4) + 0.732 × LN (CA125)

PPI and PPII were calculated using P1 and P2, respectively, as follows:

PPI = {Exp(P1) / [1 + Exp(P1)]} × 100

PPII = {Exp(P2) / [1 + Exp(P2)]} × 100

Although ROMA has better diagnostic value for OC than detection of CA125 and HE4 alone 16, it is a complicated calculation and Roche has a patent on it; therefore, it cannot be obtained using other brands. Therefore, finding more specific, sensitive, convenient, and fast tumor markers has become the focus of our research.

The contents of exosomes, including proteins and miRNAs, can be used as biomarkers in clinic settings and are effective diagnostic tools. For example, the level of exosomal miR-99a-5p derived from epithelial cells significantly increases in the serum of patients with OC, and it might serve as a target for inhibiting OC progression 17. The plasma of patients with OC contains higher levels of exosomal protein than the plasma of patients with benign tumors or healthy people 18. Because exosomes contain abundant information of origin cells, are widely distributed in various body fluids 19, and are easy to obtain, they are of great significance for the early screening, diagnosis, and prognosis evaluation of tumors. In addition, considering the stability of exosomes and the inclusion of specific proteins, we selected the tumor markers CA125 and HE4, which are associated with OC, to determine whether their levels are elevated in serum-derived exosomes.

In this study, we first identified the extracted exosomes by TEM, NTA, and flow cytometry. Experimental results showed that the extracted exosomes had the characteristics and corresponding marker proteins of exosomes. Then, to verify whether CA125 and HE4 levels are elevated in exosomes, we detected serum and exosomal CA125 and HE4 levels in the OC, NC, and non-OC groups. We found that compared with serum CA125 levels, exosomal CA125 levels significantly increased in all groups. However, exosomal HE4 levels were undetectable in any group. These results indicate that CA125 exists in serum-derived exosomes in patients with and without OC and healthy people, which is a common phenomenon rather than being unique to OC. Because CA125 is a sensitive OC marker, was the degree of increase in CA125 level in exosomes in the OC group more significant than that in the other two groups? Therefore, by calculating exosomal CA125/serum CA125 as the basis for judging the amount of increase, we compared the data of the three groups in groups of two and found that the OC group was significantly different from the other two groups in this regard. The exosomal CA125 level in the OC group increased more significantly. In conclusion, exosomal CA125 may have better diagnostic value in OC, which is worth further research.

We also selected six samples from patients with OC before and after surgical treatment to detect serum and exosomal CA125 levels. Then, we calculated pretreatment/post-treatment serum CA125 and pretreatment/post-treatment exosomal CA125 levels. If exosomal CA125 levels decreased more than serum CA125 levels after surgical treatment, then exosomal CA125 levels can be used as an indicator for dynamic monitoring of OC. However, statistical results showed that the rate of decrease of exosomal CA125 levels was not different from that of serum CA125 levels, which might be because of an insufficient sample size. In the future, we will continue to collect samples to study whether exosomal CA125 can be used as a dynamic monitoring index to monitor OC-specific changes before and after treatment.

To determine whether other tumor markers are also elevated in serum-derived exosomes, we selected serum and serum-derived exosomes from the non-OC group to measure and compare the levels of common tumor markers such as AFP, CA125, CA153, CA199 and CEA. AFP levels showed no significant difference in both serum and exosomes. Exosome CA153 levels were significantly different from serum CA153 levels, but compared with serum, CA153 level in exosomes showed a downward trend. However, CA199, CEA, and CA125 levels significantly increased in exosomes. These results indicate that other tumor marker levels increased in serum-derived exosomes, which is not specific to CA125.

Next, we assessed serum-derived exosomes of patients with OC, used pathological results as the gold standard, and drew an ROC curve using logistic regression analysis. The AUC, sensitivity, and specificity were calculated and compared with analyze the diagnostic value of serum CA125, serum HE4, exosomal CA125, exosomal/serum CA125 ratio, serum HE4+serum CA125, and serum HE4+exosomal CA125. Serum CA125 had the lowest specificity in OC compared with that in other indexes, making differential diagnosis challenging. Although HE4 specificity was higher than that of CA125, its sensitivity was poor, which might lead to the omission of OC in early diagnosis. Compared with CA125 or HE4 alone, serum HE4+serum CA125 significantly increased the AUC and sensitivity. However, the AUC, sensitivity, and specificity of exosomal CA125 significantly increased compared with those of serum CA125. The specificity of exosomal CA125 was lower than that of serum HE4, but the sensitivity was higher. Therefore, we considered combining serum HE4 with exosomal CA125, and the experimental results showed that this combination had the best effect, with significantly increased AUC and sensitivity. In addition, the exosomal/serum CA125 ratio in the OC group was higher than in the other groups. Therefore, the ROC curve of the exosomal/serum CA125 ratio was drawn to observe its diagnostic efficacy. The AUC, sensitivity, and specificity of the exosomal/serum CA125 ratio were low, so the exosomal/serum CA125 ratio is not suitable as an indicator for OC diagnosis. In conclusion, the detection of CA125 in exosomes can significantly improve the sensitivity of OC diagnosis, and serum HE4+exosomal CA125 can effectively improve the diagnostic efficiency of OC, thereby contributing to the early diagnosis of clinical OC.

ROMA has high sensitivity and specificity in OC diagnosis 20-24, and compared with CA125 and HE4 levels, ROMA is more sensitive in patients with early-stage OC 25. Therefore, during the experiment, we used exosomal CA125 to replace serum CA125 to calculate ROMA. However, we found that because the exosomal CA125 level was too high, we could not obtain the ROMA index. Whether this phenomenon suggests that the detection results of CA125 in exosomes are more valuable than ROMA in the early OC diagnosis is worth further exploration.

To identify exosomes in this study, we first performed western blotting (WB) but could not obtain the corresponding CD9 and CD63 bands. Next, we selected flow cytometry to detect CD9 and CD63, and the results showed that both CD9 and CD63 have high positive rates. We speculated that the reason that flow cytometry could detect marker proteins of exosomes but WB could not was related to the location of proteins in exosomes and the method of extracting exosomes. WB is a method of differentiating proteins of different sizes by gel electrophoresis, transferring them to membranes, and using specific antibodies to analyze a protein in a multiprotein sample. For separating proteins by gel electrophoresis, sodium dodecyl sulfate and a reductant were applied to the protein sample to cause the proteins to be linearized, so that WB detected linear protein. However, flow cytometry is a method of guiding single particles through a laser beam in a hydrodynamically focused fluid stream 26. Flow cytometry is different from WB in that it detects the conformational structure of proteins instead of the linear structure. CD9 and CD63 are marker proteins present on the membrane surface of exosomes. Protease K was added to extract exosomes by column extraction, indicating that proteinase K might damage some sites of membrane surface proteins. Therefore, protease K might also destroy some epitopes of membrane protein CD9 and CD63 antibodies, but because it has no effect on the overall spatial conformation of membrane and intramembrane proteins, the CD9 and CD63 cannot be identified using WB. Although conventional WB could not detect the presence of exosomes, the high positive rate detected using flow cytometry indicated that the sample contained more exosomes. Compared with the conventional extraction method of differential ultracentrifugation, the column extraction method has some disadvantages, such as more impurities and the inability to use WB for identification. However, column extraction is simple to operate and provides quick results, which is conducive to extensive clinical applications. In the future, serum exosomes can be rapidly extracted using column extraction for CA125 detection in clinical settings, thereby improving the accuracy of OC diagnosis and saving both time and cost.

Next, we considered why CA125 could be detected at high levels in exosomal samples using the chemiluminescence method. We speculated that this was related to the location of proteins in exosomes. As mentioned before, the extraction of exosomes using column extraction damages some membrane surface protein sites. In addition, after centrifugation of the sample, there was almost no CA125 in the waste liquid. This proved that CA125 is not destroyed or filtered during the process of extracting exosomes. We speculate that CA125 is present inside the exosome and that the exosome membrane protects it from protease digestion.

This study had a few limitations. First, the clinical sample size was insufficient; thus, there is a need to collect additional samples to verify the sensitivity and specificity of CA125 in exosomes. Second, there are no studies on the reference range and medical determination level of CA125 in exosomes. It might be inappropriate to follow the reference range and medical determination level of CA125 in serum.

In conclusion, compared with serum CA125, exosomal CA125 can be detected at higher levels, significantly improving the sensitivity of OC diagnosis. In addition, serum HE4+exosomal CA125 can significantly improve the diagnostic efficiency of OC. The application of tumor marker CA125 in serum-derived exosomes can help improve the detection rate and diagnostic accuracy of OC, providing more accurate information for clinicians to diagnose malignant tumors and evaluate prognosis.

## Figures and Tables

**Table 1:** Comparison of diagnostic efficacy of ovarian cancer (OC)-related indicators in OC

Name	AUC (%)	Sensitivity (%)	Specificity (%)
Serum CA125	0.9093	87.27	90.91
Serum HE4	0.9302	83.64	94.55
Exosomal CA125	0.9755	94.55	92.73
Exosomal CA125/serum CA125	0.6997	47.27	89.09
Combination of CA125 and HE4	0.9646	90.91	92.73
Combination of exoCA125 and HE4	0.9861	96.36	92.73
